# A Vision-Based Wayfinding System for Visually Impaired People Using Situation Awareness and Activity-Based Instructions

**DOI:** 10.3390/s17081882

**Published:** 2017-08-16

**Authors:** Eunjeong Ko, Eun Yi Kim

**Affiliations:** Visual Information Processing Laboratory, Konkuk University, Seoul 05029, Korea; goejeong85@gmail.com

**Keywords:** wayfinding system, visually impaired people, situation awareness, activity-based instruction, user trajectory recording

## Abstract

A significant challenge faced by visually impaired people is ‘wayfinding’, which is the ability to find one’s way to a destination in an unfamiliar environment. This study develops a novel wayfinding system for smartphones that can automatically recognize the situation and scene objects in real time. Through analyzing streaming images, the proposed system first classifies the current situation of a user in terms of their location. Next, based on the current situation, only the necessary context objects are found and interpreted using computer vision techniques. It estimates the motions of the user with two inertial sensors and records the trajectories of the user toward the destination, which are also used as a guide for the return route after reaching the destination. To efficiently convey the recognized results using an auditory interface, activity-based instructions are generated that guide the user in a series of movements along a route. To assess the effectiveness of the proposed system, experiments were conducted in several indoor environments: the sit in which the situation awareness accuracy was 90% and the object detection false alarm rate was 0.016. In addition, our field test results demonstrate that users can locate their paths with an accuracy of 97%.

## 1. Introduction

There are approximately 39 million legally blind people in the world, while another 246 million people have some form of significant visual impairment [1]. Among them, the number of older people is increasing due to age-related diseases such as glaucoma and diabetic retinopathy. In their daily lives, these people experience many difficulties when traversing unfamiliar environments on the way to their destination. For this type of wayfinding, it is essential to use and organize definite sensory cues from the external environment [2,3,4]. In general, sighted people construct 3D maps based on visual sensory information. In contrast, visually impaired people use different cognitive and attentional resources. As discussed in [5], people who are born blind or become blind early in life encode the sequential features of a travelled route, i.e., they create a set of instructions that denote directional changes in the route. 

To reduce the difficulties of the visually impaired and help them localize a current position and find a destination, a wide range of technologies have been developed [3,6,7,8,9,10,11,12,13,14,15,16,17,18,19,20,21,22,23,24,25,26,27,28,29,30,31,32]. The most recent research and technologies have focused on Global Positioning Systems (GPS) systems [6,7]. However, while systems using GPS sensors operate well as wayfinding aids in outdoor environments, GPS signals are often unavailable in indoor environments, which makes them inadequate for assisting people indoors. Accordingly, the goal of the present study was to develop a wayfinding system that will be effective in various indoor environments with complex illumination patterns and cluttered backgrounds such as shopping malls, hospitals, and schools.

Thus far, various solutions for indoor wayfinding have been proposed and implemented. They can be categorized as either sensor-based approaches [8,9,10,11,12,13,14,15,16,17,18,19,20,21] or vision-based approaches [22,23,24,25,26,27,28,29,30,31,32]. The former use sensors, such as Wi-Fi, RFID, and UWB sensors, to estimate a user’s current position, and the latter use images obtained from a camera and recognize visual clues, such as objects and scene texts, from the surrounding environment. Among these potential solutions, vision-based methods have received more attention from researchers, and, in particular, systems with color codes have been successfully investigated for use in various applications [26,27,28,29,30,31,32].

To help blind and visually impaired people with wayfinding, two basic functions should be supported: positioning (to localize a user’s position) and path guidance (to guide a user through a route to the target destination and the return route) [30]. To support such functions, most existing systems require the indoor structural information of buildings such as maps and building layouts. Typically, these systems obtain such information through seamless communication between a user’s mobile device and server systems, or some systems assume that a building map has been provided previously. However, in real situations, this assumption is not always true, and access to such structural information may be limited to authorized people only and is generally not common knowledge for the public. Moreover, stable communication between the server and mobile user is not guaranteed due to signal interruptions or traffic. Thus, it is necessary to develop wayfinding systems that can function in various environments regardless of the availability of maps.

For the design of a wayfinding system, we conducted an initial study on the wayfinding behaviors of visually impaired people and sighted people. We collected and analyzed the behaviors that perceive the environmental information such as the moving direction or location, and determined the next actions on the way to a given destination. In general, visually impaired people depend on a white cane to understand the environmental information. Using the white cane, they can understand the situation through detecting changes in the walls, including corners, and the ground height. When the place type changed, they determined their next actions such as finding braille signs next to the door, turning left or right according to the corner, or going up stairs. These observations signify that recognizing the current situation is essential to enabling safe travel and determining their way.

Sighted people can navigate unfamiliar indoor environments even if they do not have the structural information because they can locate the necessary information from visual clues such as objects and signs within the environment. An interesting point is that the sighted people require different types of information according to their situation. For example, when they are standing in front of a door, they need information about the room number in order to know whether it is their intended destination. For a junction or hall, they need directional information about their destination.

Based on these observations, we propose a situation-based wayfinding system that first recognizes the situation and then locates the appropriate environmental information. In this study, a situation refers to the type of place where the user is standing, and it is classified as a door, corridor, hall, or junction. In order to represent different environmental information, two types of QR code were designed: one encodes location-specific information and the other encodes directional information. These QR codes are attached according to the place type.

The proposed system was implemented on an iPhone 6, which has an embedded camera, gyroscope, and accelerometer. It consists of five processing modules: situation awareness, object detection, object recognition, user trajectory recording, and activity-based instruction. The situation awareness module is the core of the proposed system because it determines the type of scene objects to be detected according to the type of current place. For the situation awareness module, some templates that represent the respective situations are first collected. Then, a vocabulary tree is built first from templates, which are used for an effective image description and a fast comparison between images. Then, a new input image is compared with the templates using an entropy-based metric, and its situation is determined based on the most similar template. Once a situation is determined, the necessary environmental information is located. In this proposed approach, this information is represented with color QR codes [26,27,28,29,30,31,32], which require only minor modifications to the environment such as posting special signs and are widely used in real environments. Then, simple computer vision algorithms based on color and edges are applied to detect the codes on a mobile smartphone quickly and reliably. While a user is moving, their motion is computed continuously, and their routes are recorded in the user trajectory recording module, which are used to guide the return route. Finally, all processed results are conveyed to the user through activity-based instructions. These results guide visually impaired people to the destination using the user’s movement activity paths, such as walking a certain number of steps and compass directions, and the users are notified via beeping or text-to-speech (TTS) information.

To assess the validity of the proposed method, it was tested in unfamiliar indoor environments with varying illuminations and building layouts. The experimental results show that the proposed system could detect the scene objects with an accuracy of almost 100% at a distance of 2.5 m and a viewing angle of ±40°. Furthermore, it recognized the meaning of an object with an accuracy of more than 99%. In addition, to demonstrate its feasibility as a wayfinding aid for blind and visually impaired people, field tests were conducted with four users. They were all able to locate their path in real-time with an accuracy of 97%.

The reminder of the paper is organized as follows: Section 2 reviews previous work presented in the literature. Section 3 presents an overview of the proposed system. The module details are introduced from Section 4 to Section 7. The experimental results are reported in Section 8, followed by the conclusions in Section 9.

## 2. Related Work 

Over the past few years, several wayfinding systems have been developed to assist blind and visually impaired people to navigate their way through indoor environments. Giudice et al. [2] proposed four important factors that should be considered when developing electronic travel aids for blind people and visually impaired people, and then summarized the existing systems. The four factors are as follows: (1) sensory translation, which is mapping between the input and output modality that is intuitive and requires little or no training (sensors), (2) selection of information, which is important to understand what information is provided (user interface and instruction), (3) environmental conditions, which means that it can be used over a wide range of environmental conditions (environments), and (4) form and function, which means that it should be minimally intrusive (devices). Referring to these factors, we summarized the features of these systems with respect to the sensors used, their user interface, their primary functions, their target user population, and so on. Table 1 presents the existing systems for indoor wayfinding. 

### 2.1. Sensor-Based Systems vs. Vision-Based Systems

First, existing systems can be categorized into sensor-based systems and vision-based systems that use cameras. In sensor-based methods, RFIDs [8,9,10,11,12,13,14], infrared [15], ultra-wideband (UWB) [16,17], inertial sensors [18,19] and Wi-Fi [20,21] are commonly used to estimate the current position. Although these approaches work well, they have limitations. For example, RFIDs can only be sensed and read at short distances from the reader. Thus, their locations must be estimated, thereby making it difficult for blind and visually impaired people to initially locate the RFIDs [8,9,10,11,12,13,14]. In addition, pedestrian dead reckoning (PDR) systems using inertial sensors [18,19] require more computational time to improve their accuracy, because the accumulated errors increase rapidly with the travel distance. In order to compensate for this problem, Qian et al. combined the PDR algorithm with a particle filter to correct the estimated errors and guarantee the localization accuracy. However, they require additional technologies such as Wi-Fi or RFID to improve the localization accuracy. Another system using UWB-based indoor positioning was developed to provide a high level of accuracy in large open places with low installation costs. In [16,17], they used a single set of four sensors in a room with a length of less than 100 m. Their estimation errors were up to 20 cm in most locations, which is sufficiently low for application in wayfinding systems.

As an alternative, vision-based wayfinding systems have been investigated in which computer vision techniques have been used to sense the surrounding environments using scene texts, signs, and meaningful objects [22,23,24,25]. In reference [22], a body-mounted single camera was used in wayfinding for both indoor and outdoor environments. When an input image was given, environmental landmarks were identified at the position and compared with pre-established landmarks. Methods that use a stereo camera and bionic eyeglasses have also been developed in references [23,24], which recognize the current position using object recognition. In the offline stage, key objects are learned with a neural network (NN) and genetic algorithm (GA). In the online stage, the salient features are extracted from a given input image and classified as learned objects, which enable the environmental information to be recognized in the current scene. In [25], a mobile application provided location-specific information through key-frame matching. In the offline phase, the system generated list of keyframes with their distinctive camera orientations. During wayfinding, it found the closest keyframe to the current frame, computed the angular deviation between two images, and then provided the user with the current localized information. The primary advantage of these vision-based methods has been that the infrastructure or environment does not need to be modified because the objects are recognized directly as associated with specific environments. However, their disadvantages include insufficient reliability and prohibitive computational complexity. To manage this problem, a color code based system was adopted. It can operate quickly and reliably on a portable phone requiring only minor modifications to the environment [26,27,28,29,30,31,32].

**Figure 1:**
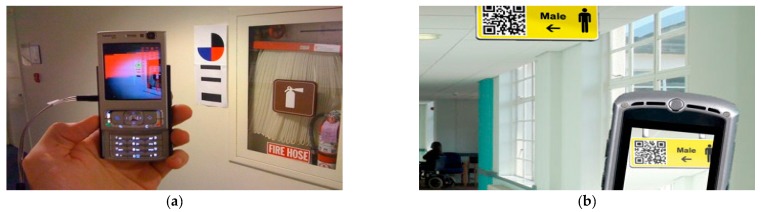
Indoor wayfinding system using color codes: (**a**) Wayfinding using color barcodes and color targets [30]; (**b**) Using quick response (QR) codes [27].

As shown in Figure 1, quick response (QR) codes and barcodes are used as labels to denote the environmental information. Among them, QR codes can contain a significant amount of information, including numbers, alphabet characters, symbols, and control codes. In practical applications, they usually represent URLs linking to webpages that explain the current environment. Such systems using color codes are effective for indoor environments and have already been proven to be practical for indoor wayfinding and shopping in grocery stores [29].

### 2.2. Assistive Functions

The core elements of wayfinding assist users by letting them know where they are, identifying their destination and providing a route for them to follow, recognizing their destination upon arrival, and safely returning them to their point of origin. To support these elements, both the positioning of the current location and the path guidance to direct users to/from the target destination should be included in a wayfinding system. 

Among the methods described in Table 1, some systems [8,12,13,15,16,19,20,22,25,28,31,32] can provide both positioning and path guidance to users, whereas others provide either positioning or path guidance. To provide both functions, most existing systems require a map to be provided or constructed. For example, they assume that building maps have been previously provided, as in references [8,12,13,15,16,19,20,22,25,28,31,32], or that the 3D building maps are constructed in real-time by extracting the landmarks from the input images and matching them to known structures [22,23,24,25]. Based on the map information, they identify the user’s current position and locate the optimal route to reach the target destination or to return to the user’s original position. However, in real situations, access to such structural information is very limited and not commonly available. Even if a map is provided, the user should obtain information through seamless communication between the mobile device and server systems; however, stable communication is not guaranteed due to signal interruptions, network traffic overload, and so on. Accordingly, it is necessary to develop wayfinding systems that can operate in various environments regardless of whether maps are available.

### 2.3. Target User Population and User Interface

During the past decade, many wayfinding systems have been developed that support the mobility of people with various impairments. The target population includes people with visual impairments which include low vision and blindness [6,7,8,9,10,11,12,13,14,15,16,17,18,19,20,21,22,23,24,25,26,27,28,29,30,31,32], people with cognitive impairments, and elderly people [33,35]. Furthermore, wayfinding has also been developed for people without impairments [34,36].

For practical applications for various user groups as mobility aids, the user interface of wayfinding systems must efficiently convey the results. Accordingly, interfaces for several types of systems have been developed for specific target user populations shown in Table 1. The types of systems can also be divided into a graphical interface using virtual reality (VR) and augmented reality (AR) techniques, haptic and audio interfaces using Braille, text-to-speech (TTS), simple audio signals or virtual sound. The former technique is commonly used in wayfinding systems designed for cognitively impaired users, whereas the latter techniques are designed for blind and visually impaired users.

In order to be effective, it is important to consider the instructions provided to the user. The instructions should be easily understood by the user. The instructions used in the existing systems can be divided into spatial language and virtual sounds. The spatial language notifies the user in the form of verbal directions (e.g., “left” or “right”) with or without degrees, which are represented as a clock reference system and cardinal directions (e.g., “Turn toward 2 o’clock” or “Turn east”). Spatial language has been widely used in the existing systems and has been proven to effectively guide the user over paths [8,9,12,16,19,23,24,25,27,28,32,37]. The virtual sounds provide spatialized sound that notifies the user using binaural sounds according to the relative distance and bearing of the waypoint with respect to the user. In [38,39], Loomis et al. performed a user study to evaluate the guidance performance between spatial language and virtual sounds. The virtual sounds exhibited better performance in terms of both guidance and user preference compared with the spatial language. However, it can partially occlude external sounds such as alarms or speech, and it can require a high computational cost to continuously track the user’s relative movements to the waypoint. 

**Figure 2:**
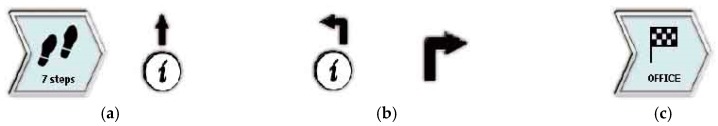
Examples of activity-based instructions: (**a**) Go straight for seven (7) steps to the information desk; (**b**) Turn left/right to the information desk; (**c**) Stop at the destination.

Among the spatial language-based approaches, activity-based instructions have been recently proposed for use in wayfinding [34]. They provide the route toward the destination divided by the minimum unit of human movement activities, and they guide a user with a specific number of steps, going up or down, turning right or left, and so on. In [34], some activity instructions are depicted in Figure 2 and all instructions are indicated using image icons. Through experiments, it was proven that activity-based instructions could reduce the mental and physical burden on the users and could provide an easier user interface with fewer errors. In this study, the activity-based instructions were redefined for the proposed system and conveyed to the user via beeping and a text-to-speech (TTS) service because blind users and visually impaired users are the target users of the proposed system.

## 3. Overview of the Proposed System

The goal of the proposed system is to guide blind and visually impaired people to and from their destination of choice in unfamiliar indoor environments to fulfill their accessibility and mobility needs. Here, the primary target user population is people with low vision or blindness. The proposed wayfinding system was implemented on an iPhone6, which has an embedded camera, gyroscope, and accelerometer. Additionally, it supports two functions: positioning and path guidance. Initially, by processing the images obtained from the iPhone camera, the proposed system first recognizes the current situation. Then, it locates scene objects and recognizes their meaning so that it can guide the user along a route to the target destination. Meanwhile, it calculates the user’s motions using the two inertial sensors and records the user’s trajectories, which are used as a guide for the return route.

**Figure 3:**
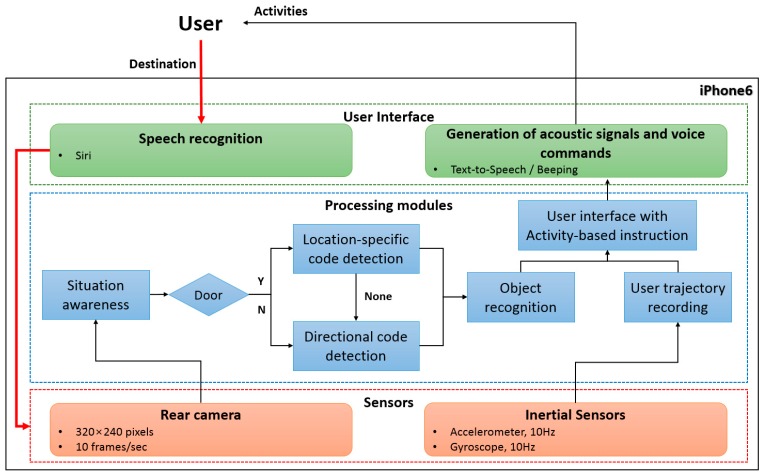
Overall architecture of the proposed wayfinding system.

Figure 3 shows the overall architecture of the proposed wayfinding system. When a user input is given to the system by speech recognition, the proposed system is activated. The information collected from the sensors are continuously processed, and the results are delivered to the user by a Text-To-Speech interface. The proposed system consists of five main modules: situation awareness, object detection, object recognition, user trajectory recording, and user interface with activity-based instructions.

In the situation awareness module, the proposed system first recognizes the user’s situation, which refers to the type of place where the user is standing. This module has an essential function in the proposed system. It can determine what environmental information is required and where the user should traverse by classifying the current types of places as corridors, doors, halls, or junctions. This enables the proposed system to function in various environments even though environmental maps are not available. For the situation awareness module, image matching techniques based on shape descriptors are used. This is discussed in detail in Section 4.

According to the situation, the required environmental information is different either requiring just location information (door) or requiring both location and directional information (corridor, hall, and junctions). Here, the environmental information is represented by QR codes. The codes were designed with green or orange quiet zones (The quiet zone helps the scanner find the leading edge of the barcode so reading can begin), which enable accurate discrimination from complex environments even when using simple color analysis. Based on the current situation and environmental information, an activity-based instruction is created and conveyed to the user. Then, human activities such as walking a certain number of steps and turning in a certain direction are obtained by calculating the distance between the user and the QR code and the viewing angle. While moving, the user’s path is continuously recorded in the user trajectory recording module, which is used to help the user locate previously visited locations such as his/her starting point.

While the user is moving, the proposed system records all processing results in a log file. Thus, it can manage the unexpected problems that can occur due to battery discharge or sensing failures. If beeping or speech signals are suddenly not generated by the proposed system, the user must stop walking and restart the system. Once the proposed system is restarted, it automatically loads the last log file in order to verify whether the recent recognition results match with the user’s destination or not; if it does not match, the proposed system determines that the previous wayfinding was not completed and asks the user if they want to continue the wayfinding for the previous destination.

## 4. Situation Awareness

People often move through unfamiliar environments. Even when people have no prior structural information for such environments, they can easily reach their destination. The reason is that sighted people use various types of visual clues that are found in indoor environments to guide them. This section describes how people use visual clues that are found in environments. Based on our observations, we defined important situations and then developed an algorithm that recognizes those situations.

### 4.1. Initial Study

**Figure 4:**
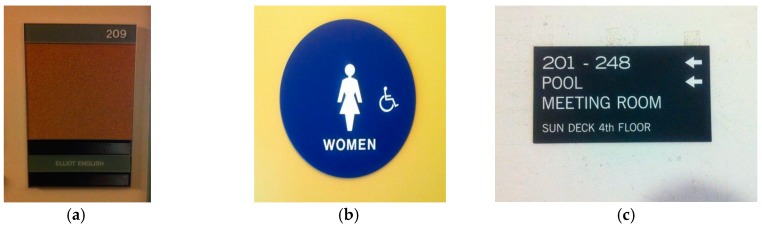
Visual clues used to guide pedestrians in real environments: (**a**) Place number; (**b**) Pictogram; (**c**) Guide sign.

To collect visual clues that are often observed in real environments, an initial study was conducted. We focused on determining which visual clues are necessary from various objects in the surrounding environment and how people use this information to guide them to their destination. In this initial study, two men and three women were selected who had expert knowledge on the structure and rooms of several buildings at our university campus. Using a digital camcorder, the users recorded useful visual clues that were found in real environments. Figure 4 shows some of the visual clues that were observed in real environments.

As seen in Figure 4, many visual clues can be observed in real environments. Although some differences exist according to the type of building, the visual clues can be divided into two groups:

- location-specific information that identifies the current position, and
- directional information for a given destination.

In addition, visual clues with positioning information were primarily observed in front of doors, while clues with directional information usually were observed in halls and corridors. For example, place numbers (Figure 4a) and pictograms (Figure 4b) were found in front of doors, whereas directional signs were found in a hall or at a junction shown in Figure 4c.

In addition, in order to analyze the needs of blind people and visually impaired people for the proposed wayfinding system, we observed their behaviors in order to understand how they interpret the environmental information around them. Yang et al. studied their wayfinding behaviors through interviews and through capturing all actions during finding their destination in unfamiliar environments [40]. In general, the visually impaired people depend on white-cane to understand environmental information. Through alternatively striking the left side and the right side with the white cane, they can perceive the location of obstacles and the available directions toward their destination. Table 2 presents the user behaviors using the white-cane to understand the environment around them. On flat ground, the visually impaired people first find the nearest wall, and then move forward following the wall. When they move around, it is possible to recognize doors or corridors through changes in the edges and shapes of the walls. On stairs or slopes, they can determine whether to go up or go down through variations in the height of the ground.

Consequently, situation information has an important function in navigation and wayfinding for both sighted people and visually impaired people. Accordingly, it was used as a foundation to develop the proposed system. To improve the mobility of blind or visual impaired people so that they can independently traverse unfamiliar indoor environments, a situation-based navigation and wayfinding system is proposed that first recognizes the type of place and then detects and recognizes the signage.

### 4.2. Definition of Situations

In this study, a situation refers to the type of place where the user is standing. The situation is categorized into one of four types: door, corridor, hall, and junction. While the first three situations (door, corridor, and hall) are self-contained, the last situation (junction) transposes one situation into another making it a combination of two or more situations. Thus, the first three situations are called primitive place types while the last situation is called a complex place type.

These types of situations have also been considered in other systems. For example, in anti-collision systems, the situations are divided into four types according to the level of difficulty in controlling a wheelchair: in front of a door, in front of an obstacle, a wall, and other situations. Then, the classification is performed based on the sensor information attached to both sides of the wheelchair. The situation is determined according to the difference between the distances measured by the sensors. This approach is unsuitable for mobile phones due to hardware issues; thus, this study proposes a novel vision-based method to recognize various situations.

### 4.3. Recognition Methods

For situation awareness, specific characteristics must be identified to distinguish the four situation types, that is, the visual patterns associated with each type of situation must be recognized. However, the same type of situation can appear significantly different in images for many reasons including cluttered backgrounds, different viewpoints, orientations, scales, lighting conditions, and so on. Thus, to manage these differences, speeded-up robust feature (SURF) is used. SURF is known as a scale and rotation invariant feature detector [41]. To establish a link between the four situation types and the SURFs, 200 images representing the four situation types were collected from various environments and used to extract the SURF local descriptors.

**Figure 5:**
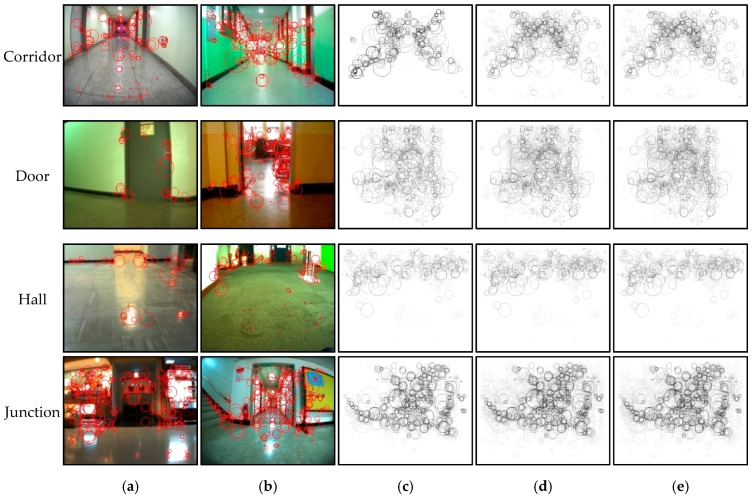
Characteristics of the SURF distributions among the situation types: (**a**,**b**) SURFs extracted from the ‘corridor’, ‘door’, ‘hall’ and ‘junction’ images; (**c**–**e**) SURF distribution accumulated with 5, 10, and 20 images, respectively.

Figure 5 shows the characteristics of the detected SURF descriptors corresponding to the four situation types. Figure 5a,b shows the SURFs overlapping the original images, while Figure 5c–e shows the accumulated SURF descriptors over 5, 10, and 20 images for each situation type. Interestingly, in Figure 5c–e, the accumulated SURFs for the corridor images revealed an X-shaped pattern shown in the top images. A rectangular-shaped pattern was observed for the door images (second image from the top). The SURFs for the hall images were crowded around the upper boundaries with a horizontal and thick line pattern in the center. A specific pattern was not detected in the junction image SURFs, which were complexly and sparsely distributed across the images. Thus, common patterns were revealed among the images that belonged to the same situation type, except for the complex junction situation. Therefore, SURF descriptors were used to index the collected sample images and to match the input image with the indexed image to recognize the current situation. A vocabulary tree, which is a very popular algorithm in object recognition, was used for the indexing [42,43]. Therefore, the module has two stages: an offline phase to build the vocabulary tree and an online phase to recognize the current situation.

#### 4.3.1. Offline Stage

First, 200 images were collected for the template data to represent the four situation types from various environments, and 20,289 SURF descriptors were extracted from the local regions in the images. Then, the extracted descriptors were quantized into visual words by the hierarchical *K*-means [42,43]. Here, *K* defines the branch factor (number of children of each internal node), not the number of clusters, and it was set at 10.

The process for the hierarchical *K*-means quantization is as follows. First, an initial clustering is performed on the 20,289 initial descriptors, thereby defining the *K* groups, where each group consists of the descriptor vectors closest to a particular cluster center. This process is performed recursively, and a vocabulary tree is built. Each node in the vocabulary tree is an associated inverted file with reference to the images containing the descriptor that corresponds to that node. Once the quantization is defined, an entropy-based weight ($w_{i}$) is assigned to each node (*i*), as follows:

(1) $$w_{i}=\ln\frac{N}{N_{i}}$$

where *N* is the number of images in the template database, and *N_i_* is the number of images in the database with at least one descriptor vector path through node *i*. Inspired by the TF-IDF scheme [42], this is used to ignore the effects of the most frequent and infrequent features (noise) in the template database.

#### 4.3.2. Online Stage

The online phase determines the most relevant images in the template database in relation to the current input image, which is calculated based on the similarity of the paths down the vocabulary tree of the descriptors from the DB images and those from the input image. According to the weights assigned to each node in the vocabulary tree, the template data (*t*) and input image (*q*) are defined as follows:

(2) $$t=\left\{t_{i}=m_{i}w_{i}\right\}.$$

(3) $$q=\left\{q_{i}=n_{i}w_{i}\right\}$$

where $m_{i}$ and $n_{i}$ are the number of descriptor vectors with a path through node *i* in the template and input image, respectively. To compute the difference between the template and input vectors, both vectors are normalized, and then, the similarity is calculated using the following dot product.

(4) $$s\left(q,\;t\right)={\left\|q-t\right\|}_{2}^{2}=2-2\sum_{\left\{fot\;all\;i|q_{i}\neq 0,\;t_{i}\neq 0\right\}}q_{i}t_{i}$$

The template image with the highest matching score is selected, and its situation type is assigned to label the current situation.

## 5. Object Detection and Recognition

It is difficult to recognize normal signage such as numbers and pictures due to their complex shapes, varieties, and distance. Due to these difficulties, color codes have been used extensively to replace normal signage [26,27,28,29,30]. Although a variety of color codes have been proposed, the QR code was chosen for the proposed system for several reasons. First, it can hold a large amount of information, including numerals, alphabet characters, symbols, and control codes. Second, the reader is freely available and can be installed on all smartphones with cameras, and it runs quickly and reliably. Accordingly, QR codes were used to represent the environmental information and then modified to increase their usability.

Unlike the existing methods that use QR codes to represent URLs [44], we used QR codes to represent numerical and alphabetic characters that indicate the positioning information such as the room numbers and signs indicating stairs and exits. Therefore, no network access is required in order to interpret the meaning of the QR codes. In addition to facilitating easier discernment of the QR codes from the background, green and orange color were used to denote the QR code quiet zones. For the minimized modification in the environment, the QR code size was set to 12 × 12 cm^2^, which was determined through experiments. The QR code was located 140 cm above the floor. 

**Figure 6:**
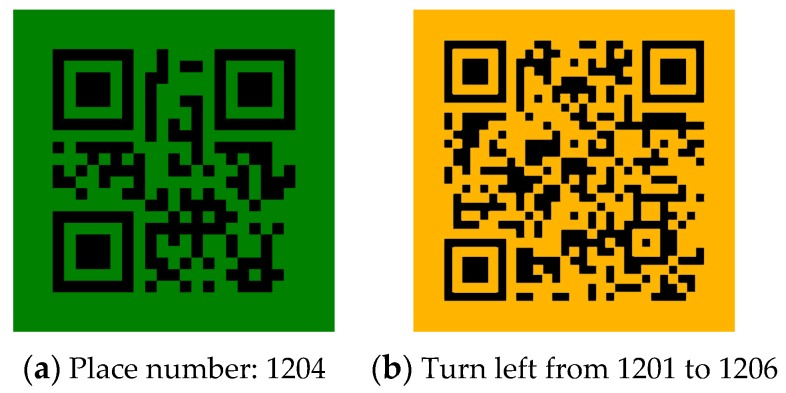
Examples of the generated QR codes and their meaning: (**a**) Location-specific code; (**b**) Directional code.

Based on the current situation, the proposed system detects different types of QR codes. The green QR codes are used to represent location-specific information, which are usually located next to the doors; the orange QR codes indicate directional information and appear in corridors, halls, and junctions. Because the standard green color (RGB (0, 255, 0)) and orange color (RGB (128, 128, 0)) in real environments appear similar to fluorescent green and orange with diffuse reflections, this study used a darker green color (RGB (0, 128, 0)) and a darker orange color (RGB (256, 186, 0)). Figure 6 presents the examples of the generated QR codes and their meanings. Figure 6a is a QR code that encodes the location-specific information: this room is 1204. Figure 6b is a QR code that encodes directional information: Turn left from room 1201 to room 1206. 

### 5.1. Object Detection

In the proposed system, the QR codes use dark green or dark orange to represent the quiet zones, and they have a square shape. Thus, they are detected by locating a green (and orange) square in a context. The process for detecting the QR codes is as follows:

(1) Preprocessing: Because time-varying illumination requires contrast adjustment, a histogram specification is used.
(2) Discretization: Each pixel in the input image is classified as green, orange or others. The color ranges are defined as follows:
  (5) $$\left(C_{r}<1.7\;\&\;C_{g}>1.5\;\&\;C_{b}>1.7\right)\&\&(H>90\;\&\;H<160)$$
  (6) $$\left(C_{r}>1.0\;\&\;C_{g}>1.0\;\&\;C_{b}<0.7\right)\&\&(H>20\;\&\;H<60)$$
(3) Labeling: Row-by-row labeling is performed on the discretized image. Then, the area and circularity are calculated from all components. These properties are used to remove noise: if the circularity of a region is larger than a predefined threshold or if its area is too small, it is considered to be noise. Thus, only components corresponding to color codes are filtered through this stage.
(4) Post-processing: After the noise filtering, the adjacent components are merged to prevent the color codes from being split.

**Figure 7:**
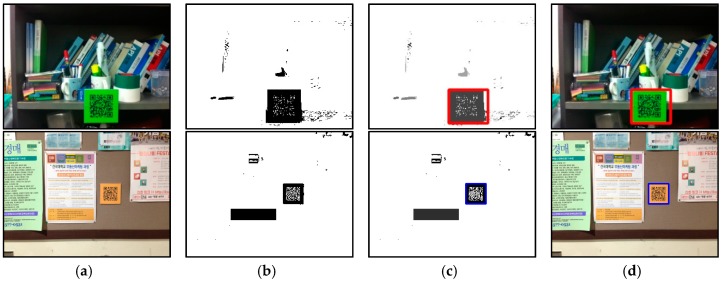
Process for QR code localization: (**a**) Input images; (**b**) Discretized results; (**c**) Labelling results; (**d**) Detected QR codes.

Figure 7 shows the process used to localize the QR codes. Figure 7a–c shows the input image, the discretized results, and the labeling. The detected regions of the QR codes are marked in red and blue; the red one indicates the detected location-specific code, and the blue one means the detected directional code, respectively. As shown in Figure 7b, the discretized image includes abundant noise, which is removed using two geometric characteristics (see Figure 7c).

### 5.2. Object Recognition

The code was attached to locations where real signage was placed in indoor environments. Once the code is localized in the detection module, the proposed system initiates the QR reader [45]. A standard QR reader can accurately recognize the detected codes within a limited range as follows: the distance from the user to the codes should be within 1 m, and the code should be perpendicular to the user. Due to these limitations, after detecting the codes, the proposed system first measures the distance between the user and the detected code and the viewing angle between them. It then verifies if the two conditions are satisfied. If not, it guides the user to approach the code and adjust his/her position so that the QR reader can read the code. The details of this process are discussed in Section 6.

## 6. User Interface with Activity-based Instructions

Recently, activity-based navigation has been proposed as an alternative to map-based navigation because it does not require a pre-installed map, and it is not dependent on absolute positioning [34]. An activity denotes the mode of human movement such as standing, walking, climbing stairs or riding an elevator. Thus, activity-based navigation guides a user to a destination using a sequence of human movement activities such as walking a certain number of steps, going up or down, and so on. The efficiency of the method in reducing the mental burden on visually impaired people and reducing navigation errors has been demonstrated previously in [34]. As mentioned above, people who go blind early and those born blind encode the sequential features of travelled route, i.e., a set of instructions that denotes the directional changes in their route. Therefore, activity-based navigation is well-suited to providing guidance information to these users.

**Figure 8:**
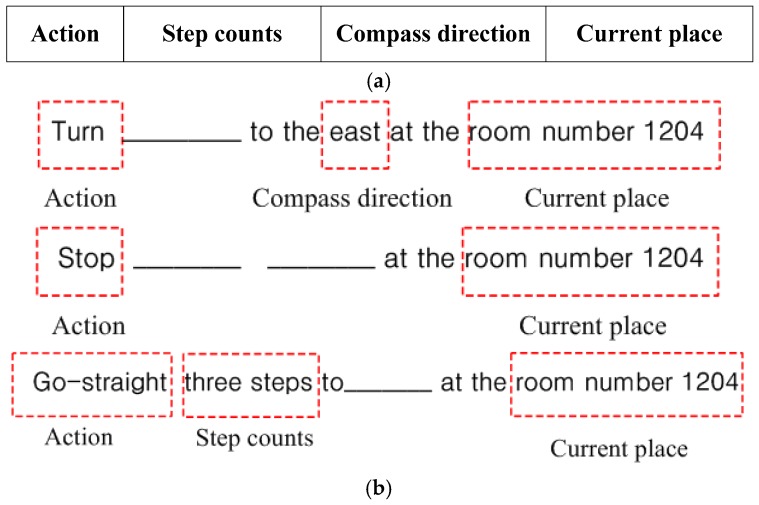
Activity-based instruction: (**a**) Structure of the instruction statement; (**b**) Three types of instructions according to the actions.

Accordingly, to convey the recognized results to users in a more efficient manner, new activity-based instructions were defined and used in the proposed system. Here, one instruction statement consists of four parameters: action, step counts, compass direction, and current place, which are shown in Figure 8a. Based on the results obtained from the situation awareness and color code recognition modules, the user action is determined. Then, the parameters necessary for the required actions are calculated by analyzing the geometric characteristics of the detected QR code. 

In addition, for the navigation system for blind users, the generated information is represented using spatial language (“turn left/right”, “go-straight,” or “stop”) or virtual sounds (i.e., the perceived azimuth of the sound indicates the target waypoint). The former is spoken to the user using a text-to-speech (TTS) service, and the latter is conveyed using beeps or sonar sounds. In the beginning of the wayfinding, spatial language is more effective for guidance to a specific direction or waypoint. However, when a cognitive load is present or accumulated while the user is moving, virtual sounds exhibited better performance than the spatial language [37,38,39]. Therefore, the proposed method combined these approaches: for the actions of “go-straight” and “stop”, the number of step counts to the destination is important. Thus, the proposed system used beeping sounds with different frequencies according to the remaining number of step counts. However, for the action of ‘turn’, the proposed system uses spatial language to convey the directional command with compass directions through the text-to-speech service. The effectiveness of the combination of these two methods was demonstrated in a field study, which is described in Section 8.2.3. Through field tests, the combined method exhibited better performance than using only speech-based spatial language.

### 6.1. Actions

In the proposed system, the possible actions are go straight, turn, and stop, each of which is determined according to several conditions such as the current situation, code detection, and viewing angle and distance between the user and the color codes. This determination process is summarized in Table 3.

When color codes are not detected, the proposed system guides the user to continue to go straight along their original direction. However, if a color code is found, it verifies whether the QR code is placed within a recognizable scope. Then, if the two QR code-reading conditions are satisfied (as specified in Section 5.2), it selects the appropriate action based on the current situation. For example, when a user is standing in a hall, junction, or corridor, it chooses the turn action (see the third row of Table 3). However, when a user is standing in front of a door, the proposed system first verifies whether the current positioning is the destination and then directs the user to stop or return to the previous route to continue his/her travel.

After determining a suitable action according to the user’s conditions, the necessary parameters for performing the respective actions should be calculated. For a go straight action, a step count is necessary to approach the detected color code or the next location. For the turn action, the compass direction is required to guide the user’s orientation for the next direction. Then, when the destination is reached, the proposed system is terminated, and it waits until a new destination is given. Accordingly, the instructions are presented in three forms according to the action type and are shown in Figure 8b.

### 6.2. Current Place

The positioning information is required for all actions. In this study, the positioning information is denoted by the current situation or current place. If explicit information is provided by decoding QR codes, the places are clearly denoted such as Room 1204, toilet, or other specific locations; otherwise, the situation such as a junction or hall is used to denote the current place. Such information is obtained through the situation awareness and object detection and recognition. 

### 6.3. Compass Direction

Here, the compass direction is described by the cardinal direction, and the possible direction is selected from the following eight orientations: {north (0°), northeast (45°), east (90°), southeast (135°), south (180°), southwest (225°), west (270°), and northwest (315°)}. Sometimes, the compass direction is clarified explicitly by the QR codes that represent directional information (see Figure 6b). However, in many cases, the compass direction is not clarified, e.g., when the detected color codes are not placed within a recognizable scope and when a user wants to return to the previous steps. To guide users in such cases, two algorithms were developed to calculate the compass direction. The first algorithm is a vision-based algorithm that was developed to calculate the compass direction based on the viewing angles between a user and the detected QR codes. The second algorithm is a sensor-based algorithm that calculates the compass direction using the difference between the user’s successive motions obtained from the gyroscope and accelerometer. In this section, only the vision-based algorithm is illustrated, and the sensor-based algorithm will be discussed in Section 7.

To calculate the viewing angles between the user and QR codes, a regular 36 × 36 grid map was used, and each cell was 5 × 5. The procedure to estimate the viewing angle between the camera and color code is as follows:

1) The regular grid map is overlaid on the detected color code.
2) For the cells allocated on both sides, the densities of the green-colored cells on the Y-axis are accumulated; each cell is denoted as DL or DR, respectively.
3) The direction of the left or right side is set by the sign of the difference between the two accumulated densities.
4) The viewing angle is determined by the difference between the two accumulated densities, i.e., |DL-DR|.

**Figure 9:**
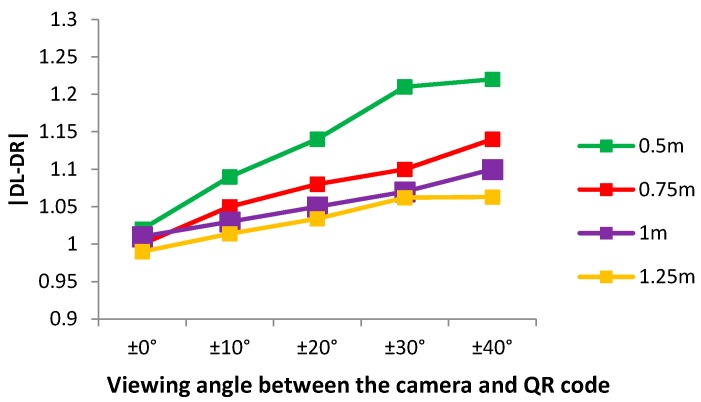
Relationship between the viewing angles and the |DL-DR| values.

Figure 9 shows that the different values as the viewing angles increase at various distances. As can be seen in this figure, these differences are directly proportional to the viewing angle between the user and the color code, that is, the difference gradually increases with larger viewing angles. 

### 6.4. Step Counts

A step count is obtained by dividing the distance to a specific position by a normal step distance. Here, a normal step distance is assumed to be 0.5 m. When QR codes are not detected and when a user is turning, the step count is fixed to 3 because the proposed system can detect objects at a distance of 2.5 m from a user. In other cases, the distance is calculated using image processing. The step count calculation is performed after estimating the viewing angle because the distance measured at the perpendicular line from the color codes is more accurate. 

**Figure 10:**
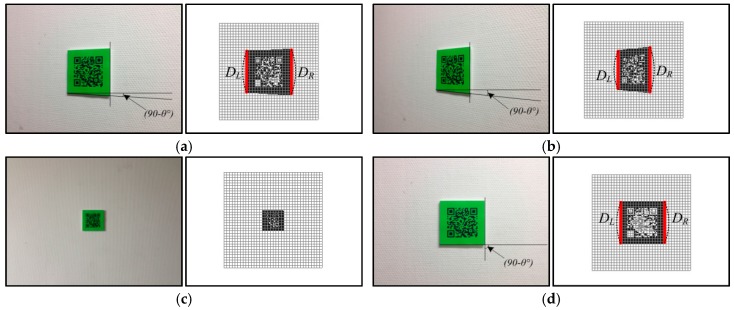
Process used to calculate the viewing angle from the camera to the color codes and the distance between them: (**a**) and (**b**) are images captured with the viewing angles of 20° and 50° at the distance of 0.5 m, respectively; (**c**) and (**d**) are images captured from the distance of 1.25 m and 0.5 m at the viewing angle of 0°, respectively.

Similar to the calculation for the compass direction, the regular grid map is first overlaid on the codes. Then, the distance is obtained by counting the number of grid cells that are mapped to the green quiet zone. Its ratio over all cells is inversely proportional to the distance, that is, the ratio gradually decreases with larger distances between the user and the color code. Figure 10 shows the color codes that are captured at several distances and viewing angles. The images in Figure 10a,b were captured from the same distance; however, they have different viewing angles of 20° and 50°, respectively. The images in Figure 10c,d were captured at a distance of 0.5 m and 1.25 m, respectively, from the user. 

To measure the viewing angles and distances from the user, the regular grid map was first overlain on the detected color codes shown in Figure 10. Then, the difference was calculated between the densities of the green-colored pixels at both ends, and the ratio of green-colored cells over all cells was counted. As shown in Figure 10, the difference in Figure 10a is larger than that in Figure 10b, and Figure 10c has a smaller ratio than that of Figure 10d.

## 7. User Trajectory Recording Module

When a user returns to his/her start point (i.e., lobby or elevator), it can cause a mental and physical burden on the user. To reduce his/her difficulties, we provide the back paths using the recorded paths. To do this, this module records a user’s trajectories until he/she arrives at his/her destination from the starting point to help him/her return to a previous location such as the starting point. Using the sensors that are already integrated into mobile phones, the paths can be constructed automatically while the user is moving. Here, two inertial sensors are used: a gyroscope and an accelerometer.

Algorithm 1 describes the algorithm used to record a user’s trajectory to the destination. All paths are recorded to the stack (S), and each path is formatted as one instruction statement. Thus, the action should be defined first, and then, the related parameters, e.g., step counts (SC), compass direction (θ), and current position (P), should be estimated. In this module, these parameters are calculated based on the sensory information.
**Algorithm 1:** The proposed trajectory recording algorithm.*Input:* Gyroscope sensor *G*, Accelerometer sensor *AC, destination D**Output*: Stack *S* that contains the set of instructions, *I*(*A*, *SC*, $\theta_{current},P)$, where *A, SC,*
θ, *P* are the variables for the action, step count, compass direction, and position, respectively.Procedure:1.**Initialize**
*A* ← null, $\theta_{previous}$, $\theta_{current}\leftarrow 0{}^{\circ}$ , *SC* ← 0, *P*($P_{x}$, $P_{y}$) ← (0,0);2.// **Determine the action type** If *AC* < 0.03, then *A* ← *Stop*3.else if $|\theta_{current}-\theta_{previous}|>15{}^{\circ}$ then *A* ← *Turn*4.else *A* ← *Go-straight*5.**//Estimate the instruction parameters according to the action type** if *A* is *Go-straight*, then *SC,*
$P_{x}$, $P_{y}$ is updated by the following equation: $\;SC\leftarrow SC+1,\;P_{x}\leftarrow SC\cdot \cos\theta_{current},\;P_{y}\leftarrow SC\cdot \sin\theta_{current}$6.else if *A* is *Turn*, then $\theta_{current}\leftarrow \theta_{previous}$7.**Push***I*(*A*, *SC*, $\theta_{current},P)$ to *S*8.**//check if the current positioning information is the destination (the positioning information is obtained by recognizing the QR codes)** if the current location is destination, then terminate9.else **Go to**
**Line 2**


Once the user arrives at the destination, the return route to the origin should be provided. The algorithm used to provide the reverse path is simple and described in Algorithm 2. As shown in Algorithm 2, the instruction that is placed on the top of the stack is conveyed to the user. Because the main users of the proposed system are blind or visually impaired, all instructions are provided verbally through text-to-speech functionality.
**Algorithm 2:** The proposed trace backward algorithm.*Input*: Stack *S* that contains the set of instructions, *I*(*A*, *SC*, θ,P), where *A, SC,*
θ, *P* are the variables for the action, step count, compass direction, and position, respectively*Output*: InstructionProcedure:1.**Pop**
*I*(*A*, *SC*, θ,P) from *S*2.if *A* is Turn, then $\theta^{\prime }$ ← (360−θ).3. 4. 5. 6. 7.**//Generate**
**the instruction**
**statement**
**according to the action type** if *A* is *Go-straight*, **Pop**
*I*(*A*, *SC*, θ,P) from *S* if *A* is *Go-straight*, then SC←SC+1 else, **Push**
*I*(*A*, *SC*, θ,P) to *S* and make *instruction* as *‘Go-straight SC steps’*
else if *A* is *Turn*, then make *instruction* as ‘*Turn to the*
$\theta^{\prime }$ else make *instruction* as ‘*Stop*’ **//Convey the instruction to the user, through Text-to-Speech (TTS) service**8.**Call** TTS (*instruction*)9.if *S* is empty, then terminate10.else **Go to**
**Line 1**


## 8. Experiments

To demonstrate the advantages of the proposed situation-based wayfinding system, two experiments were performed: an efficiency test and a field test. The efficiency test was designed to evaluate the accuracy of the five modules. In addition to demonstrating the feasibility of the proposed system in increasing the mobility of blind and visually impaired users, a field test also was designed.

### 8.1. Efficiency Test

For a quantitative performance evaluation of the proposed system, the color codes were first attached to walls inside buildings, and then, test images were collected at different times of the day and under different lighting conditions.

#### 8.1.1. Situation Awareness Results

**Figure 11:**
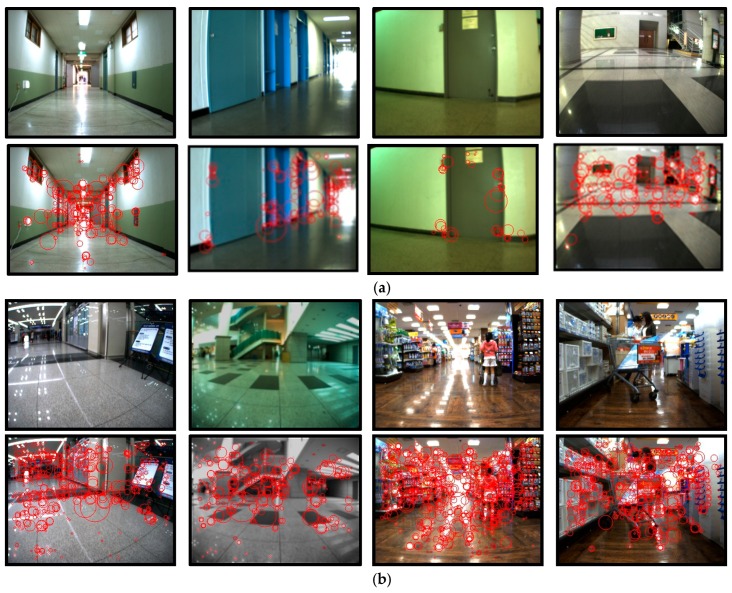
Situation awareness results: (**a**) Correctly recognized images; (**b**) Error images.

In the proposed system, the performance of the situation awareness module is crucial because it is used to select the type of QR code to be detected and it determines the actions to guide the user. In order to evaluate the accuracy of the proposed recognition method, it was tested with a total of 350 images that were captured from eight locations. Figure 11 presents some of the results from the situation awareness module in the proposed system. Figure 11a presents the SURF features overlapping the original input images, in which the scenes have cluttered backgrounds and varying degrees of illumination. These input images were compared with the template data for indexing using the vocabulary tree, and the most relevant images were selected. Figure 11b depicts examples of the images that produced errors. For robust recognition regardless of the illumination, scale, and viewpoint, the proposed situation awareness method used the SURFs to describe the images. However, this process is subject to interference from light reflections caused by surface materials. In the first two images, their situations were misclassified as corridors because the slanted lines that resulted from the reflected light created a similar complexity to the SURF descriptors for corridor images, thereby leading to the misclassification. In the second case, static objects were mistaken as walls; thus, the image was misclassified as a hall. However, the confusion between the corridor and hall classifications did not influence the performance of the proposed system because both situations required the same environmental information, i.e., directional information.

Table 4 summarizes the overall performance of the situation awareness module for various indoor environments. The average accuracy was above 90%. The accuracy in recognizing the three primitives was 93% on average. The proposed system recognized all door situations and showed perfect performance, while it had a lower precision for the corridor and hall situations. Because ‘junction’ combines two or more other primitive types and does not have distinctive features shown in Figure 5, the junction images would be misclassified as corridor or door, which does not influence the proposed system.

#### 8.1.2. Object Detection Results

For practical use, the proposed method should satisfy two requirements, which are explained in detail in reference [30]: it should detect the color codes at distances of up to 2 m in cluttered environments, and it should be robust to various degrees of illumination and cluttered backgrounds. Thus, the performance of the object detection was measured by changing the following three environmental factors:

- distance from the user (camera) to the color codes,
- viewing angle between the user and the color codes, and
- degree of illumination, e.g., direct sunlight and fluorescent light.

**Figure 12:**
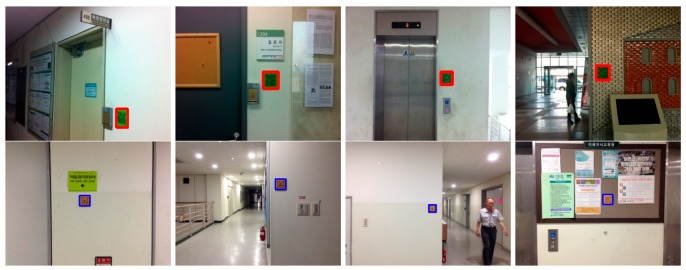
Examples of QR code localization results on real scenes.

Around 8000 images were collected using distances from 25 cm to 3 m, viewing angles from −90° to 90°, and different illuminations from direct sunlight to fluorescent light. As such, for each type of illumination, the distance was fixed at a specific value, and object detection was tested when the viewing angle was changed. Figure 12 shows examples of the object detection results in which the red rectangles represent the localized results for the color code.

**Figure 13:**
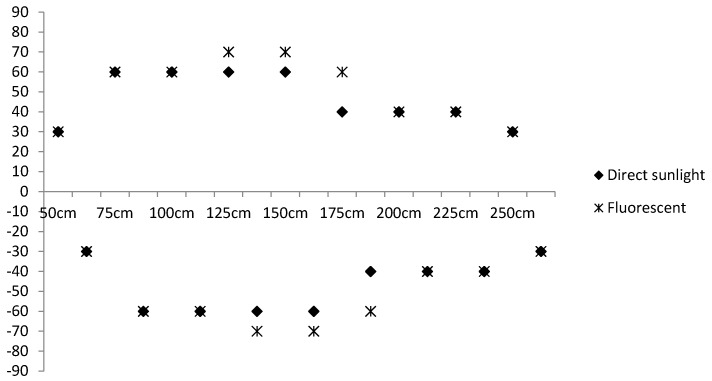
The accuracy of QR code detection in terms of the maximum detection distance (MDD) and the maximum detection angle (MDA).

Figure 13 summarizes the experimental results with the performance analyzed in terms of the maximum detection distance (MDD) and maximum detection angle (MDA). For the color codes that were perpendicularly aligned from the user, they were detected up to a maximum distance of 2.5 m. 

However, the maximum viewing distance gradually decreased as the viewing angle became further from the center. Thus, the proposed system can detect color codes with a viewing angle of 60° at a distance of 1.5 m. This detection accuracy was also affected by the degree of illumination, which showed better performance under fluorescent light than under direct sunlight. 

These results are impressive when compared with existing color code-based methods. In reference [30], a wayfinding system using color barcodes was proposed, for which color targets were designed to improve the detection distance, and these were used as the basis to denote the places of the color barcodes. By using color targets, their detection was extended to a maximum of 1.5 m. Although their detection range was extended, additional computations are involved, and the color targets, as well as color codes, should be attached to the environments. In contrast, the proposed system has a maximum detection range of 2.5 m. Accordingly it was proven that the proposed method has superior performance in accurately detecting color codes. 

**Figure 14:**
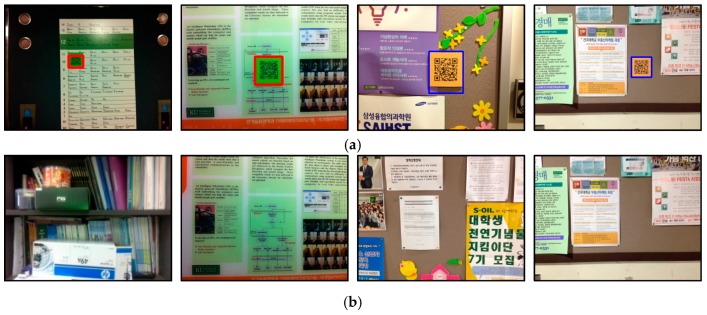
QR code localization results on complex scenes: (**a**) Images include QR codes; (**b**) Images without QR codes.

Figure 14 presents the color code localization results for images with uncluttered backgrounds and similar color compositions to the proposed QR codes. In Figure 14, the images in the first row (Figure 14a) include QR codes, while those in the second row (Figure 14b) do not. There were no false positives, and the color codes in the first row were all accurately localized.

The proposed detection method was also evaluated regarding the false positive rate (FPR) and false negative rate (FNR), which are presented in Table 5. For a distance range of 0.25 m to 3 m, the FPR was 0, and the FNR gradually increased with increasing distance. In the case in which a user turns around or moves quickly, the QR codes would be missed. However, it can be found in the next consecutive frames because the proposed system processes image streaming at 10 fps. After detecting the QR code, the proposed system asked a user to move toward the codes so that they can be more accurately recognized. After moving closer, the proposed system started the decoder, which then exhibited a recognition accuracy of 100%.

#### 8.1.3. User Trajectory Recording Results

In this study, human movements are defined in terms of action type, step count, and compass direction. Thus, there are ten basic movements including moving forward/backward and turning in the eight directions specified in Section 6.3. Furthermore, various complex movements can be produced by combining two or more basic movements. 

Accordingly, the accuracy of the user trajectory recording was evaluated using the types of movements. For these experiments, four users used the proposed system for wayfinding. First, a brief explanation of the proposed system was provided to the users, and they were shown how to use it. The users were asked to perform every movement five times, and then, the error rate was measured. The users’ moving paths were recorded, and their trajectories were compared with the real moving paths. 

**Figure 15:**
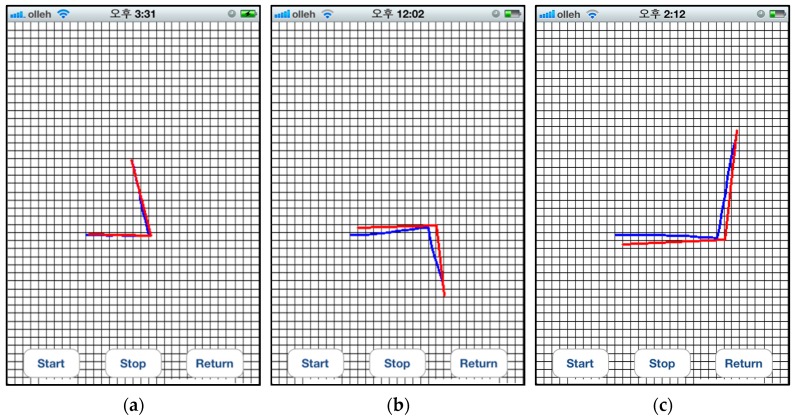
Estimation of users’ traces for complex movements: Routes generated by (**a**) User 1, (**b**) User 2, and (**c**) User 3, respectively.

The experiment was performed to evaluate the accuracy of the users’ trajectory estimation for more complex movements. In this experiment, the users were asked to return to their original starting point after reaching their destination. Figure 15 shows the results for these experiments where each cell corresponds to 0.5 × 0.5 m in real space. There are three lines: the green dotted line indicates the paths that the user used, and the two solid lines are the results from the proposed method. The solid blue line is the route from the starting point to the destination, which is estimated using the proposed user trajectory recording algorithm as shown in Algorithm 1. The red solid line is the return route for the user to return to the original starting point according to the instructions generated with the proposed backward path algorithm. 

To calculate the accuracy of the trajectory recording module, the errors between the recorded trajectories and the back paths generated by this module were calculated every second. On average, the proposed method exhibited error rates below 6% and 5% in estimating the distance and compass direction, respectively.

These error rates would be accumulative according to the increasing movements of the user in real applications. To handle this problem, a trajectory estimation method proposed in [46] was incorporated into the current system to compensate for the accumulated errors and to provide accurate return paths to the user.

#### 8.1.4. Processing Time

For practical use by blind and visually impaired users, a wayfinding system should be portable and effective in real-time. Thus, the computation time of the proposed system is a critical consideration. Table 6 presents the average time required to process a frame in the respective modules on an iPhone 6. The situation awareness module was performed at 2 s intervals when simulating real environments. On average, the proposed system required 149 ms to process a frame, followed by an average processing time of up to 13 frames/second. This confirmed that the proposed system could be used as an effective wayfinding aid in real-time situations.

### 8.2. Field Test

For practical use of the proposed system in real environments, the following three factors should be considered: real-time, performance and serviceability. Thus, several test environments were constructed, and field tests with four users were performed.

#### 8.2.1. Participants

Our goal is to provide guidance information to blind people and visually impaired people. In order to demonstrate the validity of the proposed method, we recruited participants with low vision. Thus, the field test was performed with four low vision users: three females and one male, with a median age of 26 years (range: 24–28 years). All users were able to use a smartphone in their daily lives. Table 7 provides a summary of the participants. Users 3 and 4 have significant visual impairments and cannot see objects well when they are more than an arm’s length away. 

Each user was given a basic introduction to the proposed system and was shown how to operate it. For example, it consists of entering destination via voice recognition, practices for turning each direction toward given instructions, and approaching QR code attached to the wall. They were difficult to follow the generated instruction by the proposed system, but they adapted through practices several times. These processes were performed repeatedly until they felt confidence to use the proposed system. They were asked to move from the starting point to the destination using the instructions provided by the proposed wayfinding system. In order to prevent dangerous situations that could arise, we asked one caregiver to walk alongside the users without commenting on the wayfinding during the experiments. In addition, in order to manage the hand jitter, the smartphone was fixed to the upper body of the user (approximately 130–140 cm from the floor).

**Figure 16:**
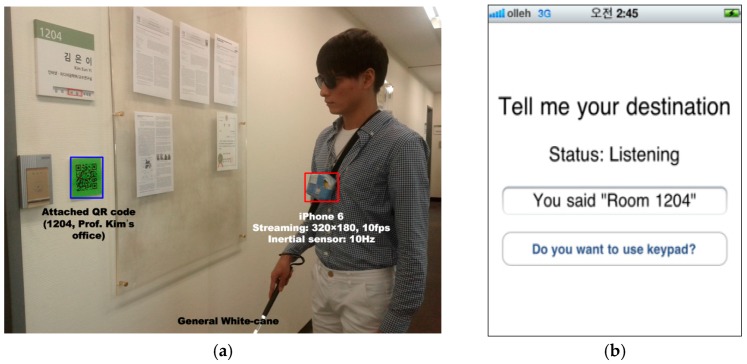
A user performing the initial tasks: (**a**) A user is moving according to the guidance by the proposed system; (**b**) Screen of the proposed wayfinding system.

Figure 16 shows a snapshot of one user using the proposed system during the field test. As seen in Figure 16b, the system required entering the destination through speech or a keypad, for example, ‘information center’ or ‘room number 1204.’ Thereafter, it continuously recognized the current situations, locates the QR codes, and interpreted them until reaching the destination. All the results that were analyzed by the proposed system were given to the users by TTS and beeping. 

Some ethical issues regarding this study should be mentioned. We complied with the principles and protocols of the Declaration of Helsinki when conducting the field test. To give the users insight into the research process, we gave them and their parents a short introduction about the research procedure and explained the informed consent for participating in the study. After the users indicated that they had examined the form and agreed to take part in the study, they signed the informed consent form. At the beginning and during the field test, the users were told repeatedly that they could terminate their participation in the study at any time.

#### 8.2.2. Test Maps

**Figure 17:**
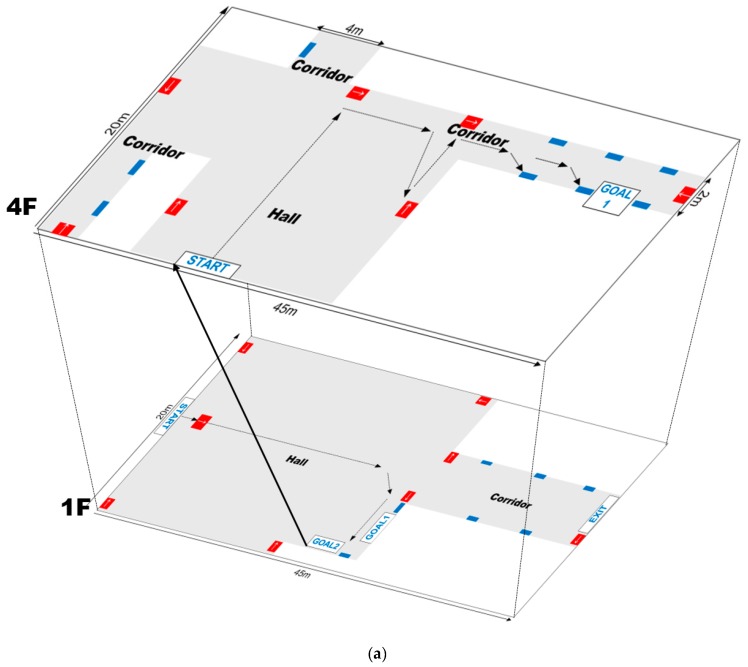

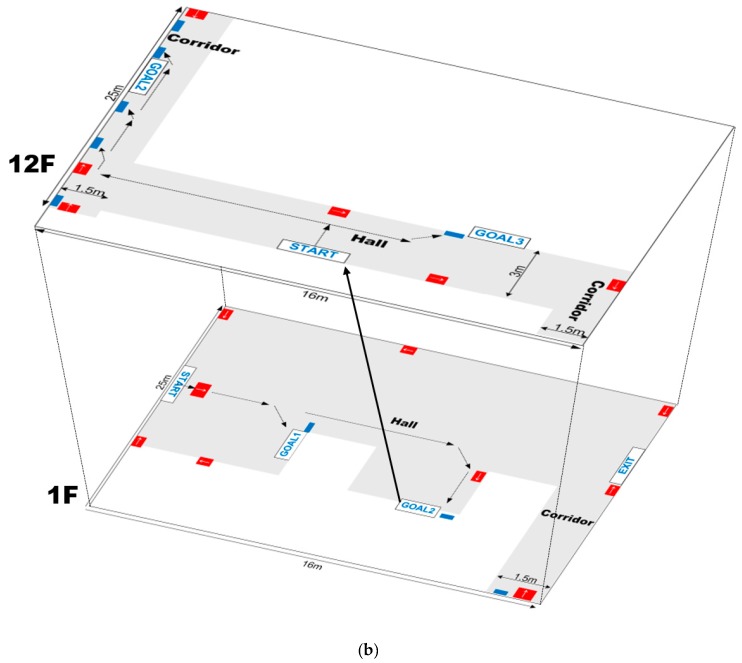
Test maps constructed for real environments: (**a**) Environmental engineering building; (**b**) New millennium building.

The goal of this research was to develop a wayfinding system that enables blind and visually impaired users to easily navigate to a specific location in unfamiliar indoor environments. In the field test, two buildings were used located on the Konkuk University campus. These buildings consisted of different building layouts. The first one was a new millennium building, which was a 14-story building with a total of 96 different rooms, and the other one is an environmental engineering building, which is a 6-story building with a total of 175 different rooms. The respective floors have almost the same structure except for the first floor, which are described in Figure 17. In these buildings, many experiments were performed with several different scenarios. Some of those scenarios are presented in this section.

The goals of the field tests consisted of: (1) arriving at room 406 on the fourth floor in the environmental engineering building, (2) finding room 1204 and (3) finding the toilet on the twelfth floor in the New Millennium building. In order to archive this, each goal was composed of two or three sub-goals. For example, the user entered the lobby on the first floor, and they had to find an information desk (sub-goal 1) and take an elevator (sub-goal 2). After getting out of the elevator, they should move toward their destination (e.g., room or toilet).

Figure 17 shows the test maps constructed for the field tests, for which the scenes contained textured and cluttered backgrounds and reflective lighting with shadows. Several QR codes were first affixed to the walls with the location and directional information. On the maps, the blue boxes indicate where location-specific codes were affixed next to doors, and the red boxes with a white arrow indicate guide codes that provide directional information. 

For the test maps, it was assumed that the users started at a predefined point and were navigating an unfamiliar environment to a predefined destination. In the test scenarios, the users were standing in the hall and had to first find the guide code to obtain directional information for the destination. Then, they had to locate the following QR codes until they reached their destination. 

When they were on the first floor (the bottom layer of Figure 17a,b), they had to first visit the information center to get the place numbers for the destinations, and then, they had to move to the target floors using the elevator. Thus, they had two goals on the first floor; finding information center and taking an elevator. Additionally, the test map in top layer of Figure 17a had one goal and the test map in top layer of Figure 17b had two goals, respectively. All these goals could be found by recognizing the QR codes of the information center, elevator, toilet, and office. 

In real environments, directional information would be not provided at junctions, which increases the difficult of wayfinding to the destination. Thus, it was assumed that guide signs were provided at every junction.

#### 8.2.3. Results

In this section, we present the experimental results of the field tests that were performed with the four users. We evaluated the performance in terms of the task time and the wayfinding errors: (1) the complete time taken from the starting point until reaching the destination was evaluated and (2) the wayfinding errors were measured through comparing users’ trajectories using the proposed system with the optimal route determined by sighted people. For this, the researchers put chalk on the shoes of the users so that their footprints and paths could be measured.

In addition, in order to evaluate the effectiveness of the user interface, the users performed the wayfinding twice for each route; the first test guided the user using only speech and the second test guided them using both beeping and speech.

**Figure 18:**
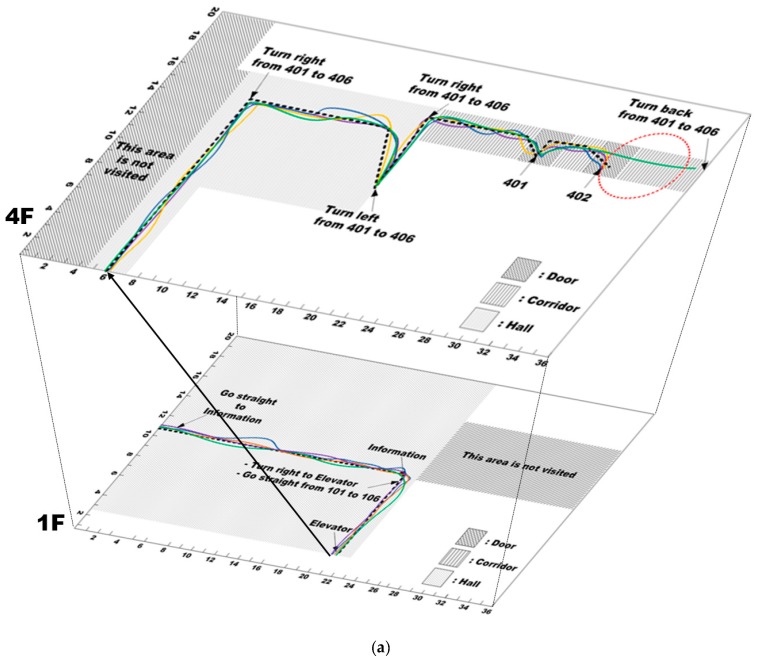

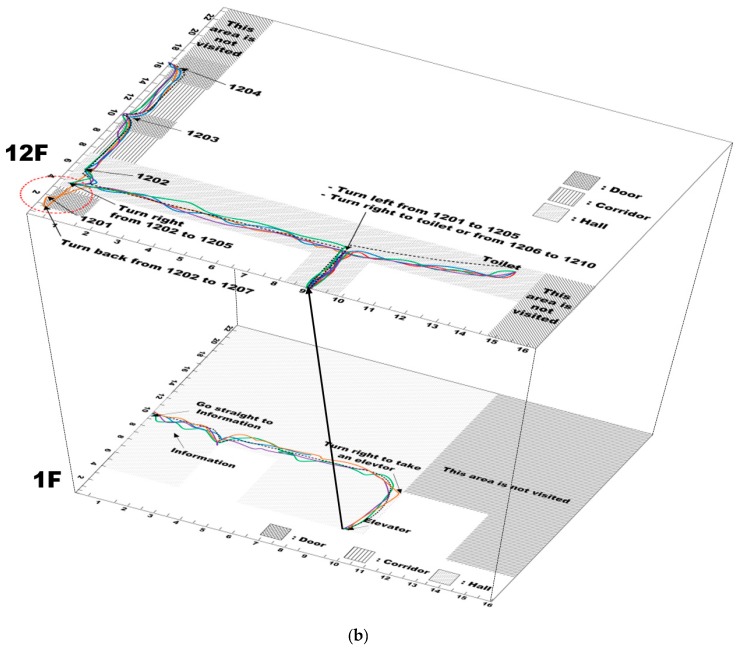
User trajectory results: (**a**) Environmental engineering building; (**b**) New millennium building. The black dotted line shows the optimal route, and the other color lines represent the traces of each user. The red circles indicate the positions where errors are occurred.

Figure 18 shows the paths of the four users when moving in the test maps shown in Figure 17. In Figure 18, the three patterns represent the respective situation types recognized by using the proposed method. As seen in the figure, the proposed system accurately recognized the situations. The black dotted line represents the optimal route created by sighted people, and the other color lines represent the paths of each user. The red circles indicate positions where errors occurred.

Despite the space complexity and lighting conditions, most users used a near optimal route in the unfamiliar indoor environment without reference points as depicted in Figure 18. Furthermore, the trajectories were similar to each other regardless of the combination of speech and beeping. However, some users made errors in their paths. In the map in the top layer of Figure 18a, User 3 made one error. As seen in Figure 17a, the width of the corridor was relatively wide, so it could be easy to miss the QR codes due to the limited FOV of the smartphone. User 1 failed to detect one QR code which denoted the target place; therefore, she continued to go straight. However, at the end of the corridor, she found the QR code that told her to turn back; thus, she could find the destination. On the map in the top layer of Figure 18b, User 4 misunderstood the instruction from the proposed system and turned in the opposite direction. However, at the end of the corridor, he found another QR code and turned back toward the destination. In addition, when the proposed system guided using only spatial language, User 4 was confused and misunderstood, which could have caused an accumulated cognitive load during a long-distance travel. Therefore, we combined the beeping information in order to reduce the cognitive load of the user and the mental demands. When the proposed system guided the users using both beeping and speech, User 4 arrived at his destination without error.

**Figure 19:**
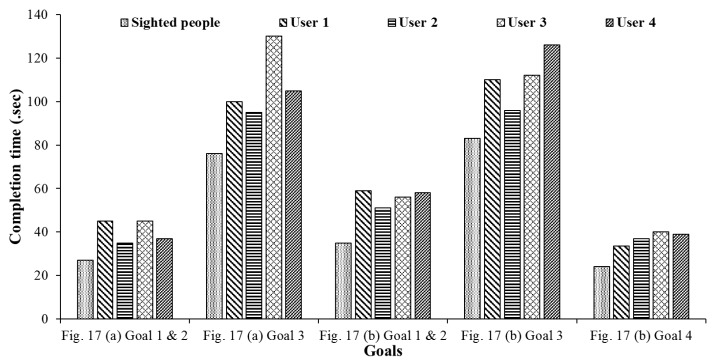
Time taken by each user to accomplish the goals (sec.).

On average, the lateral deviation relative to the optimal path was 0.5 m. Thus, even though some errors occurred, the field test results demonstrated that the proposed system had an error decision rate of less than 3% on average. Some existing systems using building maps provide the optimal route to the destination by applying the Dijkstra algorithm to preloaded maps [35]. The proposed system may provide the shortest paths to the destination; however, it does not guarantee the optimal paths because it does not use preloaded maps. Figure 19 shows the average travel time taken by the users to accomplish the goals in the respective buildings. In order to compare difficulties for approaching the attached QR code around users, we compared the average completion time obtained by two sighted students. They also followed the instructions generated by the proposed system. Two students need about 67% of completion time spent by four participants for arriving their destinations. It means that the proposed system requires training time for the visually impaired people to follow the generated instructions when they approach the QR codes. In addition, based on these data, it is clear that significant differences do not exist between the users. In the first building, the average completion time and standard variation for each goal were (40.5, 5.3) and (108.8, 15.5). In the second building, they were (56, 3.6), (111, 12.3), and (37.3, 2.9). For Goal 3 in the first building, it took User 3 a long time to reach the destination because he missed the QR codes and needed to return from the end of the corridor. Nonetheless, most users took a similar time to complete the goals. It assumed that the proposed system can be useful for the visually impaired and blind users.

#### 8.2.4. Post-test Interview Results

After finishing the field test using the proposed system, the users were interviewed in order to determine their satisfaction with the system. In order to obtain more details about their opinions, seven questions were designed based on the system usability scale [47] from ten available questions. The users were asked to rate the following seven items using one of five responses that ranged from strongly agree (5) to strong disagree (1):

- E1: I think that I would like to use this system frequently.
- E2: I thought the system was easy to use.
- E3: I think that I would need the support of a technical person to be able to use this system.
- E4: I found the various functions in this system were well integrated.
- E5: I think that most people would learn to use this system very quickly.
- E6: I thought that there was consistency in this system.
- E7: I felt very confident using the system.

**Figure 20:**
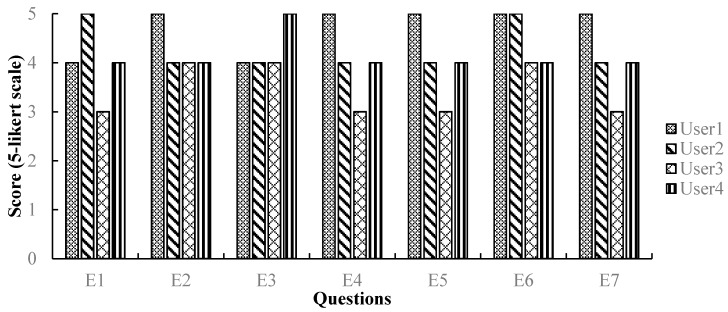
Evaluation results.

Figure 20 presents the results of the post-test interviews. As seen in the figure, most users were satisfied with the proposed wayfinding system. The users responded with average satisfaction rates of 80%, 85%, 80% for E1, E2, and E7, respectively. Regarding the proposed system’s availability in real environments, the users answered with average rates of 85% and 80% for E3 and E5, respectively. In addition, the users gave rating of 80% and 90% for E4 and E6, respectively, in the evaluation of the function and consistency of the proposed system. 

Based on the post-test interviews, some users thought that the proposed system was easy to use and helped them with their movements. Even if they needed some learning time to use the proposed system, most of the users said that they were accustomed to interacting with the proposed system after the three trials.

The overall results confirmed the feasibility of the proposed system as a wayfinding aid for visually impaired and blind users. In the field tests, the results show that the users could locate the optimal path in real-time with an accuracy of 97% and that they thought the proposed system was comfortable and efficient. Consequently, the proposed wayfinding system can effectively support visually impaired and blind users in navigating unfamiliar indoor environments.

### 8.3. Discussion

This paper has proposed a situation-based indoor wayfinding system for blind and visually impaired people. The field test results demonstrated that the proposed system can provide convenient and efficient guidance information for the users. However, it requires some improvements in providing optimal path guidance to users, increasing the localization accuracy [16,17] in cluttered environments, and extensive field testing in order to verify the generalizability of the proposed system.

First, the current system guides the user in routes based on QR code recognition results. This means that it provides limited routes according to the QR codes in the building. Thus, it cannot provide various routes such as shortcuts, detours or multiple destinations. In order to address this limitation, the map information and searching algorithm should be integrated in order to generate possible routes and to recommend the appropriate routes according to user preferences. As future work, we will integrate the GIS representation used in [16] into the current system, and employ A* algorithm [35] to generate the possible routes from given destination and GIS representation.

Second, in large open spaces, some QR codes can be hidden by pedestrians and objects, which made the proposed method miss detecting some QR codes. In order to counter this problem, we will combine with the QR code with information from nonvisual sensors such as ultra-wideband (UWB). In [17], the UWB-based system has accurate localization in the room (with sides shorter than 100 m) with a single set of four sensors, while it exhibited positioning errors up to 20 cm in most locations. In general, the installation cost for the UWBs is cheaper than RFID or Wi-Fi network-based approaches, however, it requires significant computational costs. Therefore, the proposed system can operate UWB-based localizations in cluttered environments such as junctions or halls, and switch over vision-based wayfinding in corridors and doors.

Finally, in order to demonstrate the validity of the statistical inferences, the number of users will be increased to 12–16 people. In the current study, only four people with visually impairments participated in our test. The field test should be performed by more users with greater variation in age, e.g. to include elderly people. In order to locate users with various profiles for a more extensive user study, we have been contacting official departments such as Gwangjin-gu Office (Social Welfare Division), which is a public institution in Seoul, Korea.

## 9. Conclusions

This study developed a new situation-based wayfinding system to help blind and visually impaired users recognize their location and find their way to a given destination in an unfamiliar indoor environment. The proposed wayfinding system was implemented on an iPhone 6, and it consists of five modules: situation awareness, object detection, object recognition, activity-based instruction, and user trajectory recording. 

To assess the validity of the proposed codes and wayfinding system, experiments were conducted in several indoor environments. The results show that the proposed system could detect the color codes with an accuracy of almost 100% at a distance of 2.5 m and a viewing angle of ±40°, while recognizing their meaning with an accuracy of above 99%. In addition, to confirm its real-time efficacy, field tests were performed with four users who have significant visual impairments; all the users found the optimal path in real-time with an accuracy of 97%.

A significant contribution of the proposed system over existing systems is that it does not rely on prior knowledge such as maps or 3D models of buildings by automatically predicting the outline of the buildings through situation awareness and scene object recognition. Another contribution is the development of a wayfinding system for mobile phones that are equipped with a camera and inertial sensors (i.e., gyroscope and accelerometer), which can guide users along a route to the target destination. A third significant contribution is that the proposed system has a more efficient user interface using activity-based instructions.

The proposed system needs some improvements including (1) the provision of optimal path guidance to users through combining map information with the proposed system [16,35], (2) increases in the localization accuracy through integrating the UWB technique in cluttered environments [17], and (3) verification of the generalizability of the proposed system through designing various scenarios with more varied users.

In order to fully support the mobility of blind people and visually impaired people, a system that can prevent collisions with obstacles should be incorporated into the current wayfinding system, and intensive formal validation tests should be performed with more users in order to generalize the system’s efficiency and validity. In this area of research, the previous studies of the author developed an intelligent wheelchair [48,49] and EYECANE [50,51]. The intelligent wheelchair was used for severely disabled people and it provides anti-collision maneuvers as well as a convenient user interface. EYECANE is a camera-embedded white cane that detects obstacles and find obstacle-less paths using a vision-based technique. To avoid obstacles in a more efficient manner, situation information is required, e.g., users should walk along a corridor wall, they should stop in front of a door, and so on. Thus, in future research, a technique to avoid obstacles will be developed based on situation information, and algorithms will be integrated into EYECANE. The current wayfinding system will be combined with the extended EYECANE to support safer mobility of blind and visually impaired people.

## Figures and Tables

**Table 1:** Existing systems for indoor wayfinding.

Approach		Institute	Sensors	Function	Map Usage	Target User	User Interface	Environment
Sensor-based approaches		Univ. of Catania [8]	RFID, inertial sensor	Positioning, *path guidance*	YES	The visually impaired	Speech	Indoors
		LIFC [20]	WIFI sensor network	Positioning, *path guidance*	YES	The visually impaired	Speech	Indoors
		Univ. of California-Santa Barbara [15]	Infrared	Positioning, *path guidance*	YES	The visually impaired	Spatial display	Indoors
		Univ. of California [16]	Ultra-Wide band	Positioning, *path guidance*	YES	The visually impaired	Speech	Indoors
		Univ. of Maine [19]	Accelerometers	Positioning, *path guidance*	YES	The visually impaired	Speech	Indoors
		UCEM [14]	RFID	Positioning	NO	The visually impaired	Speech	Indoors
		Universität Stuttgart [13]	RFID, GPS	Positioning, *path guidance*	YES	The visually impaired	Braille display	Indoors/Outdoors
		IEU [12]	RFID, GPS	Positioning, *path guidance*	YES	The visually impaired	Speech	Indoors/Outdoors
Vision-based ones	Scene-objects recognition	LASMEA lab [22]	CCD camera	Positioning, *path guidance*	YES	The visually impaired	Speech, Sonar sound	Indoors/Outdoors
		Univ. d'Orleans [25]	Mobile phone camera	Positioning, *path guidance*	YES	The visually impaired	Speech	Indoors
		Brigham Young University [23]	Stereo camera	Obstacle avoidance	NO	The visually impaired	Speech	Indoors
	Color-codes recognition	King Saud Univ. [22]	Mobile phone camera	Positioning	NO	The visually impaired	Speech	Indoors
		Univ. of Minnesota [32]	Infrared camera	Positioning, *path guidance*	YES	The visually impaired	Speech	Indoors
		Smart camera [27]	Mobile phone camera	Positioning	NO	The visually impaired	Speech	Indoors/ Outdoors
		UC Santa Cruz [30]	Mobile phone camera	Positioning, *path guidance*	YES	The visually impaired	Speech, beeping	Indoors
		Univ. of Malakand [28]	Mobile phone camera	Positioning, *path guidance*	YES	The visually impaired	Speech	Indoors
		Chun Yuan Christian University [33]	Mobile phone camera	Positioning, *path guidance*	YES	The Cognitively impaired	Graphical interface	Indoors
		Universidad Autónoma de Madrid [31]	Mobile phone camera	Positioning, *path guidance*	YES	The Cognitively impaired	Graphical/Verbal interfaces	Indoors
		Graz Univ. of Technology [34]	Mobile phone camera	Positioning, *path guidance*	YES	Pedestrian	Graphical interface	Indoors

**Table 2:** Wayfinding behaviors of visually impaired people.

User Behavior	Perceiving the Environments
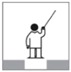	After leaving a room, they change their direction through finding the edges and shapes of the walls.
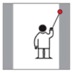	They find the nearest wall from both sides, and then follow this wall while tapping it using a white cane.
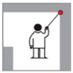	When reaching a corner, they consider the current place as a junction or court.
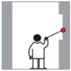	If the wall is recessed, they can assume that a door is near.
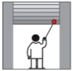	Using the height differences of the ground, they can perceive the beginning and the end of stairs.

**Table 3:** Action table.

Conditions					Action	Details
Is found any color code?	Is the distance to the detected color code less than 1 m?	Is the angle between a user and color code perpendicular?	Is the current place destination?	What is current situation?		
×	-	-	-	All	Go-straight	-
○	×	○	-	All	Go-straight	-
○	-	×	-	All	Turn	to the direction that is orthogonal to the detected QR codes
○	○	○	×	Hall, Corridor and Junction	Turn	to guided direction by directional QR codes
○	○	○	×	Door	Turn	to the direction to return back to the previous route
○	○	○	○	Door	Stop	-

**Table 4:** Confusion matrix of situation awareness (%).

	Door	Corridor	Hall	Junction
Door	100	0	0	0
Corridor	0	93	7	0
Hall	0	12	88	0
Junction	3	15	0	82

**Table 5:** Performance of QR code detection (%).

Distance (cm)	FPR	FNR
50	0	0
100	0	0
150	0	0
200	0	0
250	0	0.013
300	0	0.083

**Table 6:** Processing time (ms).

Stage	Time
Situation awareness	71
Object detection	29
Object recognition	36
Activity-based instruction	7
User trajectory recording	6

**Table 7:** Participants.

User (Age/Gender)		Ability Visual Acuity (Decimal)	Experience Mobile Phone
User1	25/Female	Low vision (0.2)	YES
User2	27/Female	Low vision (0.2)	YES
User3	28/Female	Low vision (0.15)	YES
User4	24/Male	Blind (0.01)	YES
